# MCP-1 Upregulates Amylin Expression in Murine Pancreatic β Cells through ERK/JNK-AP1 and NF-κB Related Signaling Pathways Independent of CCR2

**DOI:** 10.1371/journal.pone.0019559

**Published:** 2011-05-11

**Authors:** Kun Cai, Dongfei Qi, Xinwei Hou, Oumei Wang, Juan Chen, Bo Deng, Lihua Qian, Xiaolong Liu, Yingying Le

**Affiliations:** 1 Key Laboratory of Nutrition and Metabolism, Institute for Nutritional Sciences, Shanghai Institutes for Biological Sciences, Graduate School of the Chinese Academy of Sciences, Chinese Academy of Sciences, Shanghai, China; 2 Laboratory of Molecular Cell Biology, Institute of Biochemistry and Cell Biology, Shanghai Institutes for Biological Sciences, Chinese Academy of Sciences, Shanghai, China; University of Padova, Medical School, Italy

## Abstract

**Background:**

Amylin is the most abundant component of islet amyloid implicated in the development of type 2 diabetes. Plasma amylin levels are elevated in individuals with obesity and insulin resistance. Monocyte chemoattractant protein-1 (MCP-1, CCL2) is involved in insulin resistance of obesity and type 2 diabetes. We investigated the effect of MCP-1 on amylin expression and the underlying mechanisms with murine pancreatic β-cell line MIN6 and pancreatic islets.

**Methodology/Principal Findings:**

We found that MCP-1 induced amylin expression at transcriptional level and increased proamylin and intermediate forms of amylin at protein level in MIN6 cells and islets. However, MCP-1 had no effect on the expressions of proinsulin 1 and 2, as well as prohormone convertase (PC) 1/3 and PC2, suggesting that MCP-1 specifically induces amylin expression in β-cells. Mechanistic studies showed that although there is no detectable CCR2 mRNA in MIN6 cells and islets, pretreatment of MIN6 cells with pertussis toxin inhibited MCP-1 induced amylin expression, suggesting that alternative Gi-coupled receptor(s) mediates the inductive effect of MCP-1. MCP-1 rapidly induced ERK1/2 and JNK phosphorylation. Inhibitors for MEK1/2 (PD98059), JNK (SP600125) or AP1 (curcumin) significantly inhibited MCP-1-induced amylin mRNA expression. MCP-1 failed to induce amylin expression in pancreatic islets isolated from *Fos* knockout mice. EMSA showed that JNK and ERK1/2 were involved in MCP-1-induced AP1 activation. These results suggest that MCP-1 induces murine amylin expression through AP1 activation mediated by ERK1/2 or JNK. Further studies showed that treatment of MIN6 cells with NF-κB inhibitor or overexpression of IκBα dominant-negative construct in MIN6 cells significantly inhibited MCP-1-induced amylin expression, suggesting that NF-κB related signaling also participates in MCP-1-induced murine amylin expression.

**Conclusions/Significance:**

MCP-1 induces amylin expression through ERK1/2/JNK-AP1 and NF-κB related signaling pathways independent of CCR2. Amylin upregulation by MCP-1 may contribute to elevation of plasma amylin in obesity and insulin resistance.

## Introduction

Islet amyloid deposition is a characteristic pathologic feature of the pancreas in type 2 diabetes patients [1]. Amylin is the major component of islet amyloid deposition [2], [3]. It has been reported that the formation of pancreatic islet amyloid deposits correlates with loss of β cell mass and progressive decline of insulin secretion, suggesting a close relationship between islet amyloid deposition and the development of type 2 diabetes [1]. Amylin is mainly expressed and secreted by pancreatic β cells. Animal and human studies suggest that increased production and secretion of amylin might contribute to accumulation and aggregation of islet amyloid in pancreas. Transgenic rats with β cell overexpression of human amylin develop islet amyloid deposits which are associated with β cell death and development of hyperglycemia [4]. Therefore, to elucidate the mechanisms controlling amylin gene expression in pancreatic β cells may provide a better understanding of β cell gene expression and the pathogenesis of type 2 diabetes.

Amylin gene expression has been reported to be regulated by glucose, free fatty acids and forskolin [5]–[7]. Glucose stimulates amylin expression and secretion in a Ca^2+^ and PDX-1 dependent manner [5]. Our previous study demonstrated that Ca^2+^-PKC signaling pathways and de novo synthesized protein(s) are involved in free fatty acid-induced amylin expression [7]. Plasma amylin levels have been reported to be elevated under pathological conditions which contribute to the development of type 2 diabetes. Elevated circulating levels of amylin have been detected in obese subjects, insulin resistance and type 2 diabetes patients [8]–[11]. Pancreatic amylin mRNA and plasma amylin levels are also elevated in genetically obese, insulin-resistant rats [12]. However, the underlying mechanisms are not clear.

Obesity and insulin resistance are characterized by a chronic, systemic low-grade state of inflammation. Biomarkers of inflammation, such as TNF-α, interleukin (IL)-6, monocyte chemoattractant protein-1 (MCP-1, CCL2), and C-reactive protein, are increased in obesity, associated with insulin resistance, and predict the development of type 2 diabetes [13]–[16]. Circulating TNF-α and MCP-1 are increased in obesity and have been implicated as causative factors in obesity-associated insulin resistance and the development of type 2 diabetes [13], [17]–[20]. We recently find that TNF-α can upregulate amylin expression in pancreatic β cells [21]. In the present study, we used murine pancreatic β cell line MIN6 and pancreatic islets to examine the effect of MCP-1 on amylin expression, and further explore the underlying mechanisms.

## Results

### MCP-1 induces murine amylin expression

**Figure 1:**
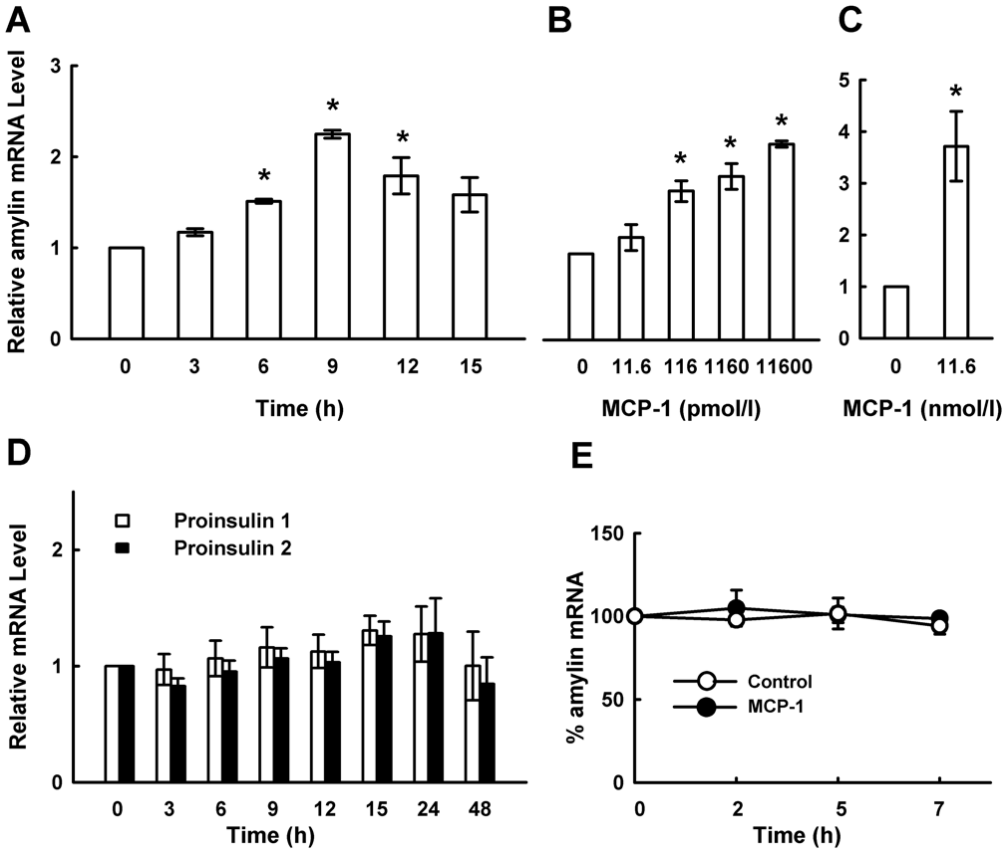
MCP-1 upregulates amylin gene expression. MIN6 cells were incubated with 11.6 nM MCP-1 for different time periods **(A, D)**, or with different concentrations of MCP-1 for 9 h **(B)**, then total RNA was extracted and examined for amylin **(A, B)** or proinsulin **(D)** mRNA level by real-time PCR. **C.** Mouse pancreatic islets were treated with 11.6 nM MCP-1 for 9 h, amylin mRNA level was examined by real-time PCR. **E.** MIN6 cells pretreated with or without 11.6 nM MCP-1 for 9 h were cultured with 5 µg/ml actinomycin D for the indicated time intervals. Amylin mRNA levels were then examined by real-time PCR. *p<0.05 vs. MIN6 cells or pancreatic islets cultured with control medium. All data are shown as mean±SD of three independent experiments.

To determine the effect of MCP-1 on amylin gene expression, murine pancreatic β cell line MIN6 was challenged with different concentrations of MCP-1 for different lengths of time, and the mRNA levels of amylin were detected by quantitative real-time PCR. As shown in Figures 1A and 1B, MIN6 cells cultured in DMEM containing 5.6 mM glucose expressed transcripts for amylin, which was significantly enhanced by MCP-1 stimulation. The minimal concentration of MCP-1 to significantly induce amylin gene expression was obtained at 116 pM with a 9 h incubation period. Consistent with the results obtained from MIN6 cells, mRNA level of amylin in murine pancreatic islets was significantly enhanced by MCP-1 after 9 h of stimulation (Figure 1C). Interestingly, the inductive effect of MCP-1 on amylin mRNA is more potent in islets than in MIN6 cells, suggesting that amylin expression in response to MCP-1 is more sensitive in islets than in transformed β cells. As amylin and insulin are co-localized in β cells and co-secreted in response to glucose [22], we then examined the effect of MCP-1 on proinsulin expression in MIN6 cells. While MCP-1 upregulated amylin mRNA levels in MIN6 cells and murine primary islets in a time-dependent manner (Figure 1A), it had no effect on proinsulin 1 and proinsulin 2 mRNA levels in MIN6 cells (Figure 1D), suggesting that MCP-1 specifically induces amylin expression in β cells.

To determine whether MCP-1-induced increase of amylin mRNA level was due to the increase of amylin mRNA stability, MIN6 cells pretreated with or without 11.6 nM MCP-1 for 9 h were cultured with 5 µg/ml actinomycin D for 2, 5, 7 h, then examined amylin mRNA levels by real-time PCR. Although MCP-1 markedly increased amylin mRNA level, there was no significant difference in the curves of mRNA decay between MCP-1 treated and control groups (Figure 1E), suggesting that MCP-1 increase amylin expression at transcriptional level.

**Figure 2:**
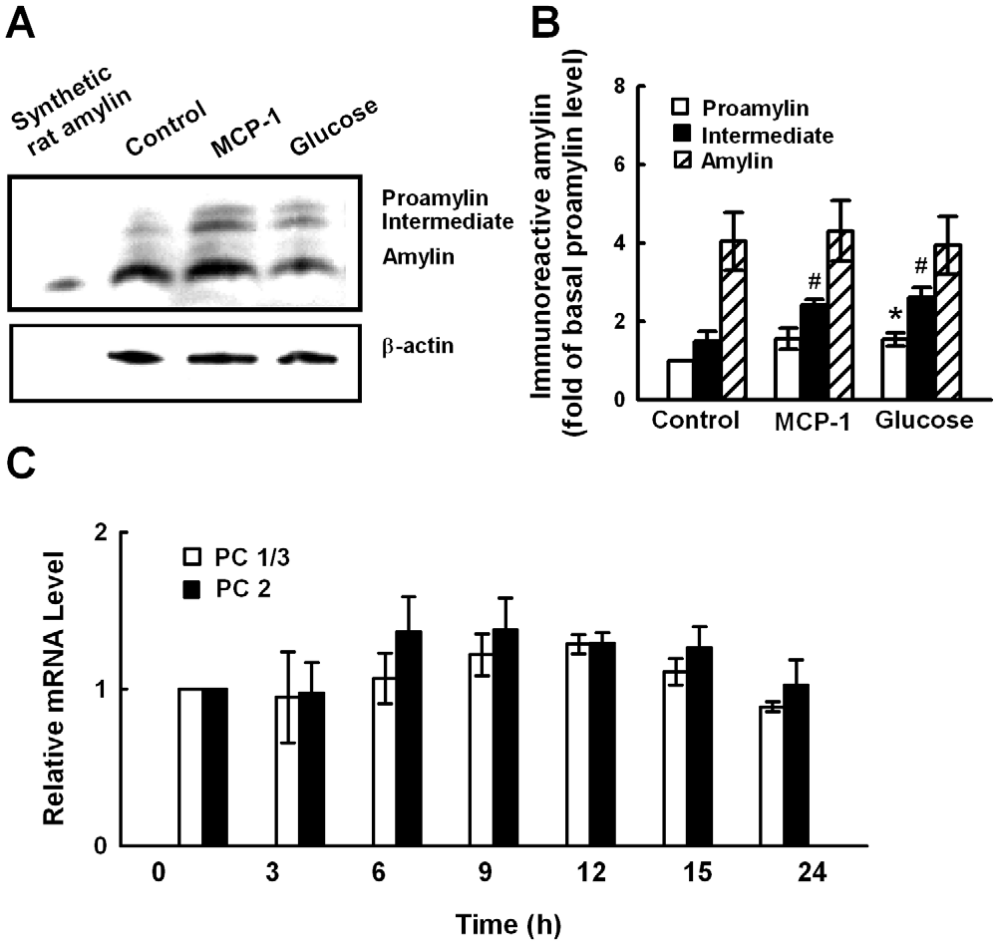
MCP-1 induces amylin protein expression. **A.** Murine pancreatic islets were cultured in control medium containing 2.8 mM glucose overnight followed by treatment with 11.6 nM MCP-1 or 16 mM glucose for 24 h. Amylin protein expression was examined by Western blot assay. A representative gel is shown, in which synthetic rat amylin was used as amylin positive control. **B.** Quantification of Western blot (**A**). *p<0.05 vs proamylin in islets cultured with control medium, ^#^ *p*<0.05 vs intermediate form of amylin in islets cultured with control medium. Mean±SD of three independent experiments. **C.** MIN6 cells were incubated with 11.6 nM MCP-1 for different time periods, then total RNA was extracted and examined for prohormone convertase (PC)1/3 and PC2 mRNA levels by real-time PCR. All data are shown as mean±SD of three independent experiments.

We next examined the effect of MCP-1 on amylin protein expression. Western blot assay showed that under resting state, mature amylin is the main form of amylin in murine pancreatic islets. Stimulation of murine islets with 11.6 nM MCP-1 or 16 mM glucose for 24 h, all significantly increased the levels of proamylin (∼8 kDa) and the intermediate form (∼6 kDa) of amylin (Figure 2A and 2B). MCP-1 had no significant effect on expressions of prohormone convertase (PC) 1/3 or PC2 (Figure 2C), which are responsible for proamylin processing [23]. These results might explain the increase of proamylin and the intermediate form of amylin by MCP-1 stimulation.

### CCR2 is not involved in MCP-1-induced amylin gene expression

**Figure 3:**
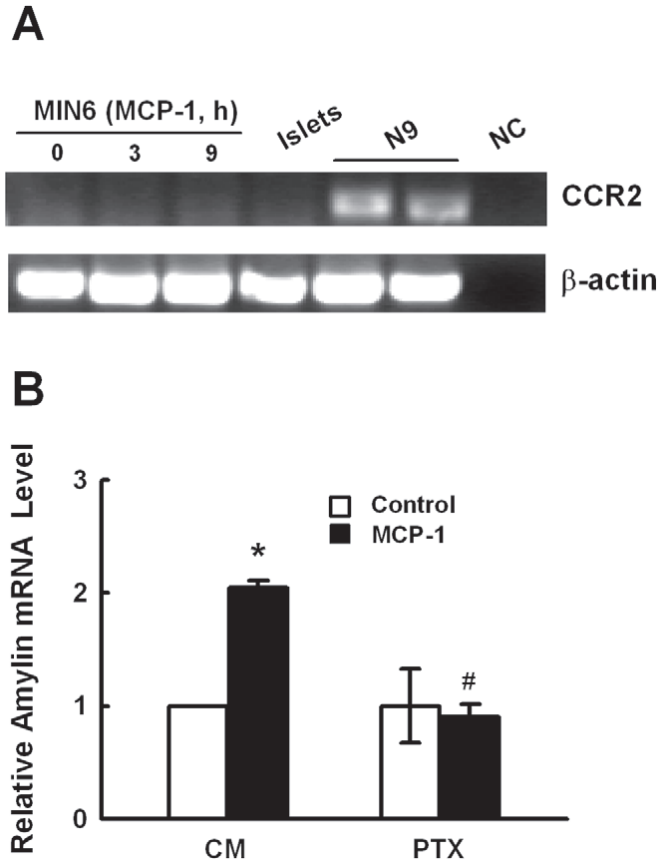
MCP-1 induces amylin gene expression independent of CCR2. **A.** CCR2 mRNA levels in MIN6 cells treated with 11.6 nM MCP-1 for different periods of time, or unstimulated islets was examined by RT-PCR. CCR2 expression in murine microglia cell line N9 was shown as positive control. NC: negative control without cDNA template. **B.** MIN6 cells were incubated with control medium (CM) or 0.5 µg/ml pertussis toxin (PTX) for 1 h, then stimulated with 11.6 nM MCP-1 for 9 h and examined for amylin expression by real-time PCR. *p<0.05 vs cells cultured with CM. ^#^ *p*<0.05 compared with cells treated with MCP-1 alone. Mean±SD of three independent experiments.

As CCR2, a Gi-coupled receptor, is the only known receptor that functions at physiologic concentrations of MCP-1. We examined if MCP-1 induces amylin expression through CCR2. Surprisingly, CCR2 mRNA was not detectable in both MIN6 cells and murine islets, and treatment of MIN6 cells with 11.6 nM MCP-1 for up to 9 hours had no effect on CCR2 expression (Figure 3A). However, the induction of amylin by MCP-1 was inhibited by pretreatment of the cells with *pertussis* toxin (Figure 3B), suggesting that alternative Gi-coupled receptor(s) mediates the inductive effect of MCP-1.

### AP1 activation by ERK1/2 or JNK is involved in MCP-1-induced amylin gene expression

**Figure 4:**
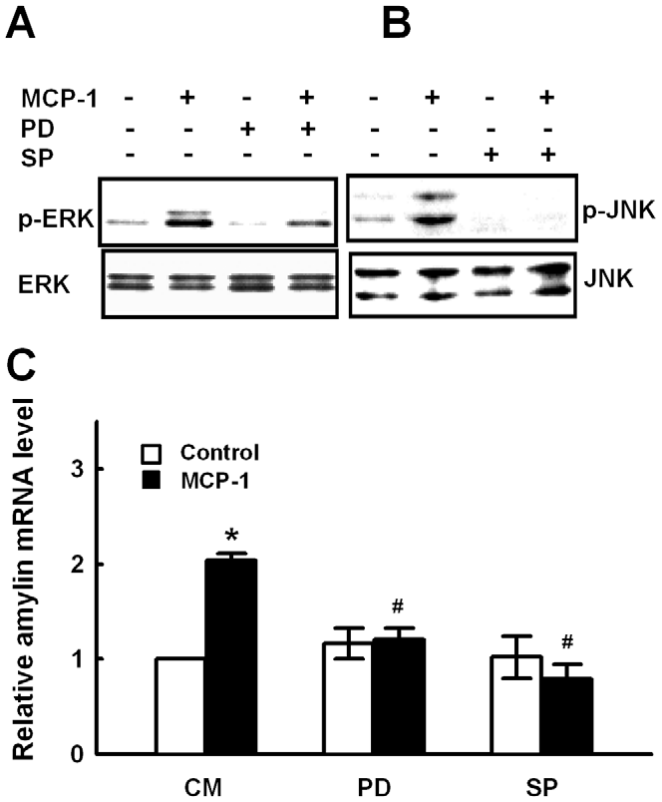
ERK1/2 and JNK signaling pathways are involved in MCP-1-induced amylin gene expression. **A–B.** MIN6 cells pretreated with 30 µM PD98059 (PD) or 50 µM SP600125 (SP) for 2 h were stimulated with 11.6 nM MCP-1 for 5 min. ERK1/2 or JNK phosphorylation was examined by Western blot. The experiments were performed at least three times and representative results are shown. **C.** MIN6 cells were incubated with control medium (CM), 30 µM PD98059 (PD) or 50 µM SP600125 (SP) for 1 h, then stimulated with 11.6 nM MCP-1 for 9 h and examined for amylin expression by real-time PCR. *p<0.05 vs cells cultured with CM. ^#^ *p*<0.05 compared with cells treated with MCP-1 alone. Mean±SD of three independent experiments.

It has been reported that MCP-1 activates extracellular signal-regulated kinase (ERK) and c-Jun NH2-terminal kinase (JNK) MAPK in endothelial cells and monocytes/macrophages [24]–[26]. So we tested if MAPK activation was involved in MCP-1-induced amylin gene expression. As shown in Figures 4A and 4B, treatment of MIN6 cells with 11.6 nM MCP-1 stimulated rapid phosphorylation of ERK1/2 and JNK. Pretreatment of MIN6 cells with PD98059 (MEK1/2 inhibitor) and SP600125 (JNK II inhibitor) significantly inhibited MCP-1-induced ERK1/2 and JNK phosphorylation, respectively. We observed that pretreatment of MIN6 cells with PD98059 and SP600125 both significantly inhibited MCP-1-induced amylin gene expression (Figure 4C). PD98059 and SP600125 at tested concentrations had no effect on cell viability as examined by MTT assay (data not shown). These results indicate that MCP-1 upregulates amylin gene expression through activation of ERK1/2 and JNK related signaling pathways.

**Figure 5:**
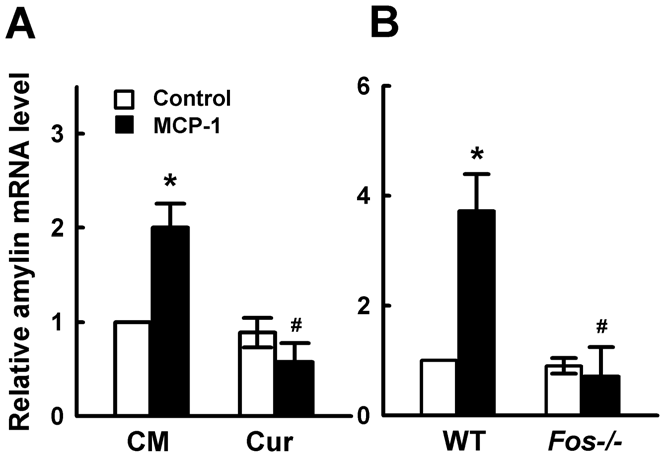
MCP-1 stimulates amylin gene expression through activation of AP1. **A.** MIN6 cells pretreated with control medium (CM) or 10 µM curcumin (Cur) for 1 h were stimulated with 11.6 nM MCP-1 for 9 h, then amylin mRNA levels were examined by real-time PCR. *p<0.05 vs cells cultured with control medium. ^#^ *p*<0.05 compared with cells treated with MCP-1 alone. **B.** Mouse pancreatic islets isolated from wild type (WT) or *Fos* -/- mice were treated with 11.6 nM MCP-1 for 9 h, amylin mRNA level was examined by real-time PCR. *p<0.05 vs. islets from WT mice cultured with control medium. ^#^ *p*<0.05 vs. islets from WT mice in response to MCP-1. All data are shown as mean±SD of three independent experiments.

Transcription factor activator protein-1 (AP1) has been reported to mediate MCP-1 induced inflammatory activation of human tubular epithelial cells and smooth muscle cell proliferation [27], [28]. When we analyzed murine amylin gene with AliBaba 2.1 program (http://wwwiti.cs.uni-magdeburg.de/grabe/alibaba2.), several AP1 binding sites were revealed to exist in the promoter region. To examine whether AP1 is involved in MCP-1-induced amylin gene expression, we pretreated MIN6 cells with curcumin, an inhibitor of AP1 [29], and then we challenged these cells with 11.6 nM MCP-1. We found that curcumin significantly inhibited amylin mRNA upregulstion by MCP-1 (Figure 5A), indicating AP1 is involved in MCP-1-induced amylin gene expression. Curcumin at tested concentration had no effect on cell viability as examined by MTT assay (data not shown). As curcumin has been reported to regulate various molecules [30], the possibility of the involvement of other curcumin targets in amylin upregulation by MCP-1 can not be ruled out. Fos protein is a component of AP1 which is a dimeric protein complex [31]. We further examined the effect of MCP-1 on amylin expression in pancreatic islets isolated from *Fos* knockout mice and got negative results (Figure 5B). These results confirm that AP1 plays an essential role in the induction of amylin gene expression by MCP-1.

**Figure 6:**
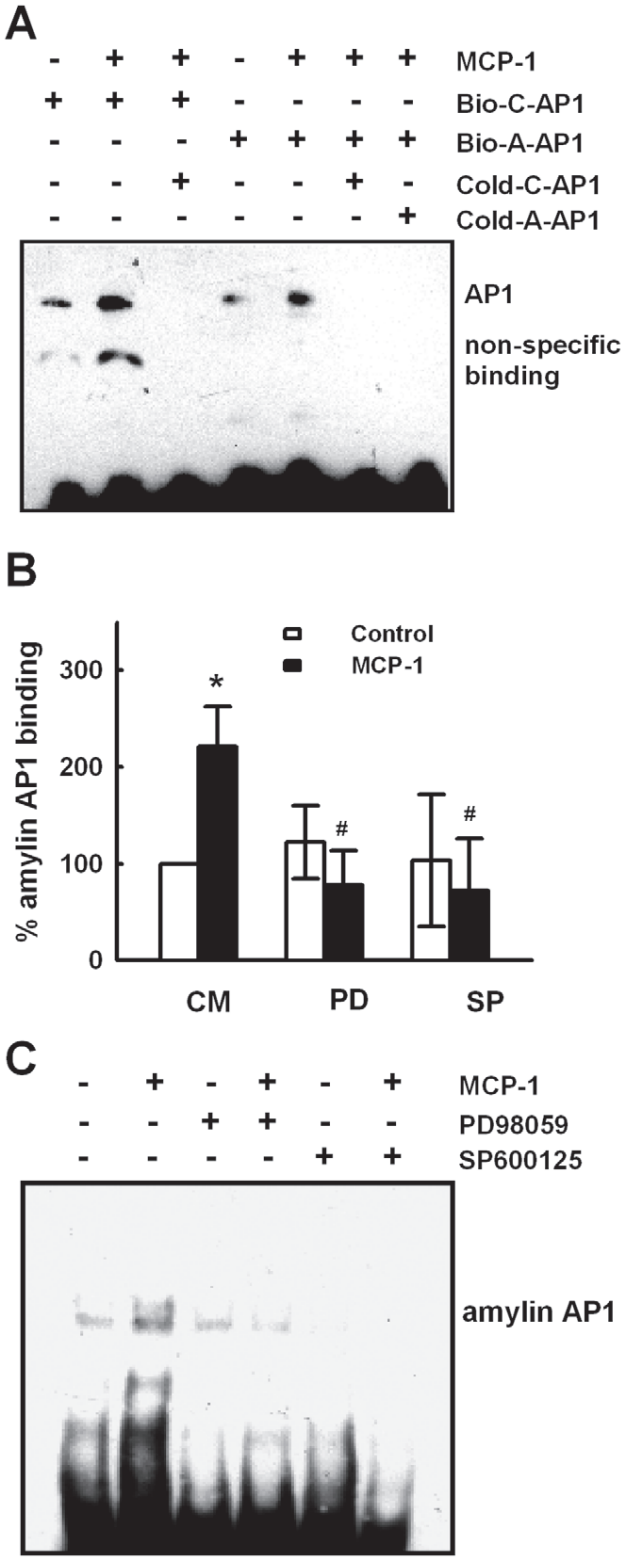
MCP-1 activates AP1 through ERK1/2 and JNK. **A.** MIN6 cells were treated with or without 11.6 nM MCP-1 for 2 h, the nuclear protein was extracted and applied for EMSA using biotin-labeled amylin AP1 probe (Bio-A-AP1 probe) or consensus AP1 probe (Bio-C-AP1 probe). 100-fold of unlabeled consensus AP1 probe (cold-C-AP1) or amylin AP1 probe (cold-A-AP1) was used as competitor. The experiments were performed at least three times and representative results are shown. **B.** MIN6 cells pretreated with 50 µM SP600125 (SP) or 30 µM PD98059 (PD) for 1 h were stimulated with 11.6 nM MCP-1 for another 2 h. The nuclear protein was extracted and applied for EMSA using biotin-labeled amylin AP1 probe. *p<0.05 vs cells cultured with control medium. ^#^ *p*<0.05 compared with cells treated with 11.6 nmol/l MCP-1 alone. Mean±SD of three independent experiments. **C.** A representative gel of B is shown.

ERK and JNK have been reported to be upstream molecules of AP1 [31]. We then tested if MCP-1 activates AP1 through ERK1/2 and JNK in pancreatic β cells. EMSA assay with a consensus AP1 probe (C-AP1), or an amylin AP-1 probe (A-AP1) which contains the AP1 binding sequence at the promoter region (-1574/-1568) of murine amylin gene, showed that MCP-1 significantly increased the binding activity of AP1 in MIN6 cells (Figure 6A, B). In MCP-1-stimulated cells, both excess unlabeled C-AP1 probe and A-AP1 probe could compete for amylin AP1 binding, and excess cold C-AP1 could compete for consensus AP1 binding (Figure 6A), suggesting that the A-AP1 probe is specific, and MCP-1 induced AP1 binding to the promoter region of amylin gene. The increased amylin AP-1 activity induced by MCP-1 was significantly inhibited by pretreatment of MIN6 cells with JNK inhibitor SP600125, or MEK1/2 inhibitor PD98059 (Figure 6B, C). Taken together, these results suggest that MCP-1 induces amylin gene expression in β cells through ERK1/2-AP1 and JNK-AP1 pathways.

### MCP-1 induces amylin gene expression through NF-κB related pathways

**Figure 7:**
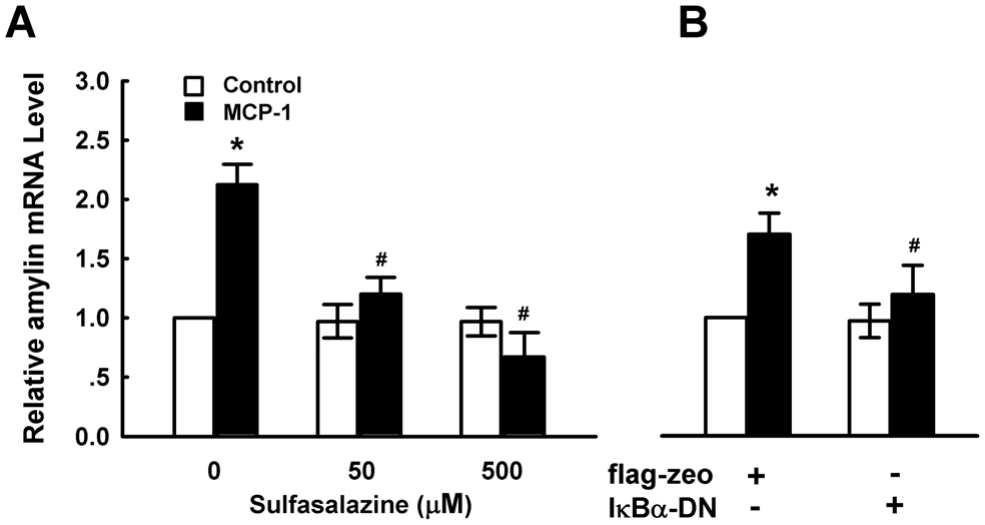
Involvement of NF-κB signaling pathway in MCP-1-induced amylin gene expression. **A.** MIN6 cells pretreated with control medium or different concentrations of sulfasalazine for 1 h were stimulated with 11.6 nM MCP-1 for 9 h, then amylin mRNA levels were examined by real-time PCR. *p<0.05 vs cells cultured with control medium. ^#^ *p*<0.05 compared with cells treated with MCP-1 alone. **B.** MIN6 cells transiently transfected with *IκB*α dominant-negative construct (*IκB*α-DN) or control vector flag-zeo for 36 h were treated with control medium or 11.6 nM MCP-1 for another 9 h, amylin mRNA levels were examined by real-time PCR. *p<0.05 vs. flag-zeo-transfected cells cultured with control medium. ^#^ *p*<0.05 vs. flag-zeo-transfected cells in response to MCP-1. Mean±SD of three independent experiments.

Transcription factor nuclear factor-κB (NF-κB) is another signaling molecule involved cell activation by MCP-1 [27], [28]. Thus we checked if NF-κB was involved in MCP-1 -induced amylin gene expression in pancreatic β cells. Pretreatment of MIN6 cells with sulfasalazine (NF-κB inhibitor) [32] significantly inhibited MCP-1-induced amylin gene expression (Figure 7A). As sulfasalazine at tested concentrations had no effect on cell viability (data not shown), these results suggest that NF-κB is involved in amylin upregulation by MCP-1. To further confirm that NF-κB activation is involved in the induction of amylin expression by MCP-1, we transfected MIN6 cells with an *IκB*α dominant-negative construct (*IκB*α-DN) containing a S32A and S36A substitutions [33] or control vector flag-zeo. Overexpression of *IκB*α-DN in MIN6 cells (Figure 7B) significantly attenuated MCP-1-induced amylin gene expression, suggesting an essential role of NF-κB in the upregulation of amylin expression by MCP-1.

## Discussion

In the current study, we studied the effect of MCP-1 on amylin gene expression in both mouse pancreatic β cell line MIN6 and pancreatic islets. We found that MCP-1 upregulated amylin mRNA and protein levels but had no effect on amylin mRNA stability. We further demonstrated that MCP-1 induced amylin gene expression through ERK1/2/JNK-AP1 pathways and NF-κB related signaling pathways which are independent of CCR2.

Islet amyloid is a pathological hallmark of the pancreatic islet present in a substantial proportion of individuals from all ethnic groups with type 2 diabetes [34]–[36]. Studies in spontaneous islet amyloid formation in macaques and domestic cats have shown that amyloid forms in islets before fasting hyperglycemia, and the extent of amyloid deposition is associated with both loss of β cell mass and impairment in insulin secretion and glucose metabolism, suggesting a causative role for islet amyloid in the islet lesion of type 2 diabetes [37]. Mechanistic studies have demonstrated that amylin can inhibit β cell insulin secretion [38], induce β cell apoptosis [37], [39], [40], and cause insulin resistance [41], [42] in both *in vitro* and *in vivo* systems. In addition, prefibrillar assemblies of human amylin can bind and disrupt lipid bilayers and form ion-permeable pores, leading to destabilization of the intracellular ionic environment [43], [44]. Therefore, elucidation of the mechanisms involved in amylin expression and deposition will not only contribute to the understanding of the pathogenesis of type 2 diabetes but also provide novel strategies for the treatment and prevention of this disease.

Insulin resistance is an important contributor to the pathogenesis of type 2 diabetes, and obesity is a risk factor for its development. Recent data have revealed that the plasma concentrations of inflammatory mediators, such as TNF-α, MCP-1 and IL-6, are increased in the insulin resistant states of obesity [13]. Increased circulating levels of MCP-1 and amylin have been detected in obese and type 2 diabetes patients [8]–[11], [14], [15], [45], [46]. High MCP-1 levels contribute to diabetes risk independently of previously-described clinical, metabolic and immunological risk factors [16]. In vitro studies showed that MCP-1 could directly induce insulin resistance in adipocytes and skeletal muscle cells [47], [48]. Animal studies have demonstrated that increase of MCP-1 expression in adipose tissue contributes to the macrophage infiltration into this tissue and insulin resistance [49], [50], while systemic elevation of MCP-1 is sufficient to induce systemic insulin resistance irrespective of adipose tissue inflammation [51]. Our results demonstrated that MCP-1 induced amylin expression in pancreatic β cells at both mRNA and protein levels, indicating that in addition to directly induce insulin resistance, MCP-1 may indirectly contribute to insulin resistance by upregulating amylin expression. The upregulation of amylin gene expression by MCP-1 may be an important link between chronic inflammation and insulin resistance.

Our studies showed that MCP-1 induced murine amylin expression in MIN6 cells and pancreatic islets at transcription level. The inductive effect of MCP-1 is more potent in islets than in MIN6 cells. As MIN6 cells and murine islets don't express MCP-1 receptor CCR2 but the induction of amylin by MCP-1 can be blocked by *pertussis* toxin, the inductive effect of MCP-1 may be mediated through other Gi-coupled receptor(s) than CCR2. Supporting our results, other researchers also reported the absence of detectable CCR2 mRNA in murine β-cell line and islets [52]. MCP-1 has been reported to induce cell responses of mouse astrocytes and human aortic smooth muscles with no detectable CCR2 [53], [54], and to induce the expression of tissue factor in aortic smooth muscles isolated from CCR2 knockout mice through alternative Gi-coupled receptor(s) [55]. By using a series of biochemical and molecular biology methods with specific inhibitors for MAP kinases, AP1, NF-κB, dominant-negative constructs for *IκB*α, and islets from Fos-/- mice, we demonstrated that MCP-1 induced amylin expression in murine pancreatic β cells through JNK/ERK1/2-AP1 and NF-κB pathways. AliBaba2 program analysis revealed that there are only AP1 binding sites but not NF-κB binding sites existing in the promoter region of mouse amylin gene. Therefore, NF-κB activation may play an indirect role in murine amylin upregulation by MCP-1.

Amylin is synthesized in β cells as a precursor molecule, proamylin. Production of mature amylin from proamylin is a two-step process, initiated predominantly by cleavage at its COOH terminus by the prohormone convertase (PC)1/3, followed by cleavage of the resulting NH2-terminally extended intermediate by PC2 [23]. Our studies showed that MCP-1 increased proamylin and the intermediate form of amylin in murine islets. We found that MCP-1 stimulated amylin expression but had no significant effect on PC1/3 and PC2 expression, which may lead to the upregulation of proamylin and the intermediate form of amylin but not the mature form of amylin. In addition to mature amylin, NH2-terminally extended proamylin is also a component of islet amyloid [56]. It has been proposed that defects in the processing, sorting, and/or secretion of proamylin by β cells may initiate amyloid formation [1], [57]–[59]. Amylin precursor upregulation by MCP-1 may contribute to amylin elevation and amyloid formation. Pancreatic amyloid is found in humans but not in rodents. This has been attributed to the amino acids in some regions of human amylin which are easy to form aggregate [60]. Both human and rodent amylin are secreted in response to glucose, free fatty acids and food intake [5], [7]. Whether the amylin secretion pathway in human and rodent are different upon MCP-1 stimulation or under pathological conditions, and the effects of MCP-1 on human proamylin processing and amylin deposition need further investigation.

Taken together, our studies demonstrate that MCP-1 induces amylin gene expression in murine β cells through JNK/ERK1/2-AP 1 and NF-κB related signaling pathways. Our results and available evidence suggest that in addition to direct involvement in insulin resistance, MCP-1 may play an important role in overexpression of amylin in obesity and insulin resistance which are causally linked to the development of type 2 diabetes.

## Materials and Methods

### Materials

MCP-1 was purchased from Peprotech Inc. (Rocky Hill, NJ, USA). Pertussis toxin, PD98059, SP600125, sulfasalazine, and curcumine were from Calbiochem (La Jolla, CA, USA). Type V collagenase was from Sigma Aldrich (Louis, MO, USA). Ficoll 400 was purchased from Amersham Pharmacia Biotech (Piscataway, NJ, USA). DMEM was from Gibco BRL (Burlington, Ontario, Canada). Lipofectamine 2000 was from Invitrogen (Carlsbad, CA, USA). Unless otherwise stated, all other reagents were purchased from Sigma Aldrich.

### Pancreatic islet preparation and cell culture

Pancreatic islets were isolated from C57/BL6 mice (from Shanghai SLAC Laboratory Animal Company, China) or *Fos* knockout mice [61] by type V collagenase digestion followed by Ficoll 400 gradient separation, as described previously [62]. Islets were cultured in DMEM containing 5.6 mM glucose, 10% FBS, 100 U/ml penicillin and 100 g/ml streptomycin. Batches of 100 and 250 islets were used for RNA extraction and Western blot assay, respectively. All experiments using animals were in accordance with the ‘Principles of laboratory animal care’ (NIH publication no. 85–23, revised 1985; http://grants1.nih.gov/grants/olaw/references/phspol.htm), and were approved by the Institutional Animal Care and Use Committee, Institute for Nutritional Sciences, Chinese Academy of Sciences (Permit No. 2009-AN-01). MIN6 cells, a mouse pancreatic β cell line, were cultured in DMEM containing 5.6 mM glucose, 10% FBS, and antibiotics as above in a humidified atmosphere at 37°C with 5% CO_2_.

### RNA extraction, PCR and real-time PCR

Total RNA was extracted from MIN6 cells or mouse pancreatic islets using the Trizol reagent (Invitrogen, Carlsbad, CA, USA) and depleted of contaminating DNA with RNase-free DNase according to manufactures’ instructions. cDNA was synthesized from 2 µg RNA with M-MuLV reverse transcriptase and random hexamer according to manufacturer's instructions (Fermentas, Burlington, Ontario, Canada). Reverse-transcribed cDNA in triplicate samples were checked for target mRNA level by PCR or quantitative real-time PCR with Power SYBR Green PCR master Mix (Applied Biosystems Inc, Warrington WAI4SR, UK) on ABI Prism 7500 sequence detector (Applied Biosystems Inc, Foster City, CA, USA). Primers used in the experiment were: murien CCR2: 5′-ATAAGGGCTCTTGTTTGATCTTTFCC (sense), 5′-TGGCTATTCCATATACACCTTTCCC (antisense) [63]; murine amylin: 5′-CAGCTGTCCTCCTCATCCTC (sense), 5′-GCACTTCCGTTTGTCCATCT (antisense); murine β-actin: 5′-CAACGAGCGGTTCCGAT (sense); 5′-GCCACAGGATTCCATACCCA (antisense); murine proinsulin 1: 5′-TCTTCTACACACCCAAGTCCCG (sense), 5′-CTCCAACGCCAAGGTCTGAA (antisense); murine proinsulin 2: 5′-CTTCTTCTACACACCCATGTCCC (sense), 5′-CCAAGGTCTGAAGGTCACCTG (antisense); murine prohormone convertase (PC) 1/3: 5′- ACATGGGGAGAGAATCCTGTAGGCA (sense), 5′-CATGGCCTTTGAAGGAGTTCCTTGT (antisense); murine PC2: 5′- TTGATGCAGGTGCCATGGTGAA (sense), 5′-ACTTGTCAAAGCCCACCTTGGAGT (antisense). Amplification of the target cDNA was normalized to β-actin expression. Relative levels of target mRNA expression were calculated using the 2^-ΔΔC^_T_ method.

### Western Blotting

Phosphorylation of ERK1/2 or JNK was examined as described previously [64]. Briefly, MIN6 cells were stabilized in KRB buffer for 2 h followed by stimulation with MCP-1 for 5 min. The cells were then lysed with cold RIPA lysis buffer containing 150 mM NaCl, 5 mM EDTA, 1% Triton X-100, 0.1% SDS, 100 mM Tris (pH 8.0), 10 mM NaF, 1% deoxycholic acid, 1 mM PMSF, 1 mM sodium vanadate, 1 mM DTT, 10 µM Aprotinin and 4 µM leupeptin. The cell lysates were centrifuged at 12,000 rpm for 20 min to remove insoluble materials, and the concentration of protein was determined by Bradford assay. Protein were electrophoresed on 10% SDS-PAGE gel, transferred onto polyvinylidene difluoride (PVDF) membrane (Millipore Corporation, Bedford, MA) and probed with antibodies against phosphorylated ERK1/2 (Santa Cruz Biotechnology, CA) or JNK (Cell Signaling Technology, Beverly, MA), followed by incubation with a horseradish peroxidase-conjugated secondary antibody. Immunoreactive bands were detected by Supersignal West Pico chemiluminescent substrate (Pierce, Rockford, IL, USA) and X-Omat BT film (Eastman Kodak Company, Rochester, New York, USA). The membranes were stripped and re-probed with antibody against ERK1/2 or JNK (Cell Signaling Technology, Beverly, MA) to ensure equal loading.

To examine the expression of amylin protein by murine islets, islet proteins (10–15 µg) were electrophoresed on a 15% polyacrylamide gel using Tris-tricine buffer [7], transferred onto PVDF membrane, and probed with anti-rat amylin antiserum (T-4145, Peninsula Laboratory, Belmont, CA, USA), or anti-β-actin monoclonal antibody (Sigma Aldrich, Louis, MO, USA), followed by incubation with a horseradish peroxidase-conjugated secondary antibody.

### Transient transfection

MIN6 cells were transfected with dominant-negative plasmid or control vector using SuperFect Transfection Reagent (Qiagen, Valencia, CA) according to the manufacture's instruction. Thirty-six hours after transfection, the cells were stimulated with MCP-1 for 9 hours, and amylin mRNA expression was then detected by real-time PCR. IκBα-DN, a dominant-negative IκBα expressing plasmid, and its control vector flag-zeo were a kind gift from Dr. R. Lin (McGill University, Montreal, Canada).

### Electrophoretic mobility shift assay (EMSA)

MIN6 cells were cultured in medium without FBS for 12 h, then were treated with or without various inhibitors for 1 h followed by 11.63 nM MCP-1 for another 2 h. The nuclear proteins were prepared with NE-PER Nuclear and Cytoplasmic Extraction Reagents (Thermo, Rochford, USA). The protein concentration was determined using Bradford assay. EMSA was performed with LightShift Chemiluminescent EMSA kit (Pierce Chemical Co., Rockford, USA). Briefly, 20 µg nuclear proteins were pre-incubated with binding buffer for 10 min at 4°C and then incubated with biotin-labeled AP1 probe for another 20 min at room temperature. For competition experiments, a 100-fold excess of unlabelled doubled-stranded AP1 oligonucleotides was added to the binding reaction. DNA-protein complexes were analyzed by electrophoresis in 4% polyacrylamide gels. Complexes were transferred to a nylon membrane (Pierce Chemical Co., Rockford, IL, USA) and crosslinked to the membrane using a hand-held UV lamp equipped with 254 nm bulbs. Migration of the biotinylated oligonucleotides and their complexes was detected by chemiluminescence followed by exposure of the membrane to X-ray films. The oligonucleotide sequences of AP1 probes were: amylin AP1 probe: 5′-AAGAGCTTGAGTCACACAAGA-3′ ; consensus AP1 probe: 5′-CGCTTGATGACTCAGCCGGAA-3′.

### Statistical analysis

Results are expressed as means±SD of at least three independent experiments. Statistical analysis was performed using ANOVA for time-course and dose-response, and Student's t test for other data.

## References

[1] Marzban L, Park K, Verchere CB (2003). Islet amyloid polypeptide and type 2 diabetes.. Exp Gerontol.

[2] Cooper GJ, Willis AC, Clark A, Turner RC, Sim RB (1987). Purification and characterization of a peptide from amyloid-rich pancreases of type 2 diabetic patients.. Proc Natl Acad Sci U S A.

[3] Westermark P, Wernstedt C, Wilander E, Hayden DW, O'Brien TD (1987). Amyloid fibrils in human insulinoma and islets of Langerhans of the diabetic cat are derived from a neuropeptide-like protein also present in normal islet cells.. Proc Natl Acad Sci U S A.

[4] Matveyenko AV, Butler PC (2006). Beta-cell deficit due to increased apoptosis in the human islet amyloid polypeptide transgenic (HIP) rat recapitulates the metabolic defects present in type 2 diabetes.. Diabetes.

[5] Macfarlane WM, Campbell SC, Elrick LJ, Oates V, Bermano G (2000). Glucose regulates islet amyloid polypeptide gene transcription in a PDX1- and calcium-dependent manner.. J Biol Chem.

[6] Ding WQ, Holicky E, Miller LJ (2001). Glucose and forskolin regulate IAPP gene expression through different signal transduction pathways.. Am J Physiol Endocrinol Metab.

[7] Qi D, Cai K, Wang O, Li Z, Chen J (2010). Fatty acids induce amylin expression and secretion by pancreatic beta-cells.. Am J Physiol Endocrinol Metab 2010;.

[8] Sanke T, Hanabusa T, Nakano Y, Oki C, Okai K (1991). Plasma islet amyloid polypeptide (Amylin) levels and their responses to oral glucose in type 2 (non-insulin-dependent) diabetic patients.. Diabetologia.

[9] Eriksson J, Nakazato M, Miyazato M, Shiomi K, Matsukura S (1992). Islet amyloid polypeptide plasma concentrations in individuals at increased risk of developing type 2 (non-insulin-dependent) diabetes mellitus.. Diabetologia.

[10] Enoki S, Mitsukawa T, Takemura J, Nakazato M, Aburaya J (1992). Plasma islet amyloid polypeptide levels in obesity, impaired glucose tolerance and non-insulin-dependent diabetes mellitus.. Diabetes Res Clin Pract.

[11] Reinehr T, de Sousa G, Niklowitz P, Roth CL (2007). Amylin and its relation to insulin and lipids in obese children before and after weight loss.. Obesity (Silver Spring).

[12] Huang HJ, Young AA, Koda JE, Tulp OL, Johnson MJ (1992). Hyperamylinemia, hyperinsulinemia, and insulin resistance in genetically obese LA/N-cp rats.. Hypertension.

[13] Lee YH, Pratley RE (2005). The evolving role of inflammation in obesity and the metabolic syndrome.. Curr Diab Rep.

[14] Christiansen T, Richelsen B, Bruun JM (2005). Monocyte chemoattractant protein-1 is produced in isolated adipocytes, associated with adiposity and reduced after weight loss in morbid obese subjects.. Int J Obes (Lond).

[15] Kim CS, Park HS, Kawada T, Kim JH, Lim D (2006). Circulating levels of MCP-1 and IL-8 are elevated in human obese subjects and associated with obesity-related parameters.. Int J Obes (Lond).

[16] Herder C, Baumert J, Thorand B, Koenig W, de Jager W (2006). Chemokines as risk factors for type 2 diabetes: results from the MONICA/KORA Augsburg study, 1984-2002.. Diabetologia.

[17] Lang CH, Dobrescu C, Bagby GJ (1992). Tumor necrosis factor impairs insulin action on peripheral glucose disposal and hepatic glucose output.. Endocrinology.

[18] Cheung AT, Ree D, Kolls JK, Fuselier J, Coy DH (1998). An in vivo model for elucidation of the mechanism of tumor necrosis factor-alpha (TNF-alpha)-induced insulin resistance: evidence for differential regulation of insulin signaling by TNF-alpha.. Endocrinology.

[19] Sell H, Eckel J (2007). Monocyte chemotactic protein-1 and its role in insulin resistance.. Curr Opin Lipidol.

[20] Sell H, Eckel J (2009). Chemotactic cytokines, obesity and type 2 diabetes: in vivo and in vitro evidence for a possible causal correlation?. Proc Nutr Soc.

[21] Cai K, Qi D, Wang O, Chen J, Liu X (2011). TNF-α acutely upregulates amylin expression in murine pancreatic beta cells.. Diabetologia.

[22] Kahn SE, D'Alessio DA, Schwartz MW, Fujimoto WY, Ensinck JW (1990). Evidence of cosecretion of islet amyloid polypeptide and insulin by beta-cells.. Diabetes.

[23] Marzban L, Trigo-Gonzalez G, Verchere CB (2005). Processing of pro-islet amyloid polypeptide in the constitutive and regulated secretory pathways of beta cells.. Mol Endocrinol.

[24] Werle M, Schmal U, Hanna K, Kreuzer J (2002). MCP-1 induces activation of MAP-kinases ERK, JNK and p38 MAPK in human endothelial cells.. Cardiovasc Res.

[25] Cambien B, Pomeranz M, Millet MA, Rossi B, Schmid-Alliana A (2001). Signal transduction involved in MCP-1-mediated monocytic transendothelial migration.. Blood.

[26] Sodhi A, Biswas SK (2002). Monocyte chemoattractant protein-1-induced activation of p42/44 MAPK and c-Jun in murine peritoneal macrophages: a potential pathway for macrophage activation.. J Interferon Cytokine Res.

[27] Viedt C, Dechend R, Fei J, Hänsch GM, Kreuzer J (2002). MCP-1 induces inflammatory activation of human tubular epithelial cells: involvement of the transcription factors, nuclear factor-kappaB and activating protein-1.. J Am Soc Nephrol.

[28] Viedt C, Vogel J, Athanasiou T, Shen W, Orth SR (2002). Monocyte chemoattractant protein-1 induces proliferation and interleukin-6 production in human smooth muscle cells by differential activation of nuclear factor-kappaB and activator protein-1.. Arterioscler Thromb Vasc Biol.

[29] Hahm ER, Cheon G, Lee J, Kim B, Park C (2002). New and known symmetrical curcumin derivatives inhibit the formation of Fos-Jun-DNA complex.. Cancer Lett.

[30] Zhou H, Beevers CS, Huang S (2011). The targets of curcumin.. Curr Drug Targets.

[31] Young MR, Yang HS, Colburn NH (2003). Promising molecular targets for cancer prevention: AP-1, NF-kappa B and Pdcd4.. Trends Mol Med.

[32] Weber CK, Liptay S, Wirth T, Adler G, Schmid RM (2000). Suppression of NF-kappaB activity by sulfasalazine is mediated by direct inhibition of IkappaB kinases alpha and beta.. Gastroenterology.

[33] Zhao T, Yang L, Sun Q, Arguello M, Ballard DW (2007). The NEMO adaptor bridges the nuclear factor-kappaB and interferon regulatory factor signaling pathways.. Nat Immunol.

[34] Zhao HL, Lai FM, Tong PC, Zhong DR, Yang D (2003). Prevalence and clinicopathological characteristics of islet amyloid in chinese patients with type 2 diabetes.. Diabetes.

[35] Clark A, Saad MF, Nezzer T, Uren C, Knowler WC (1990). Islet amyloid polypeptide in diabetic and non-diabetic Pima Indians.. Diabetologia.

[36] Höppener JW, Ahrén B, Lips CJ (2000). Islet amyloid and type 2 diabetes mellitus.. N Engl J Med.

[37] Hull RL, Westermark GT, Westermark P, Kahn SE (2004). Islet amyloid: a critical entity in the pathogenesis of type 2 diabetes.. J Clin Endocrinol Metab.

[38] Ohsawa H, Kanatsuka A, Yamaguchi T, Makino H, Yoshida S (1989). Islet amyloid polypeptide inhibits glucose-stimulated insulin secretion from isolated rat pancreatic islets.. Biochem Biophys Res Commun.

[39] Huang CJ, Haataja L, Gurlo T, Butler AE, Wu X (2007). Induction of endoplasmic reticulum stress-induced beta-cell apoptosis and accumulation of polyubiquitinated proteins by human islet amyloid polypeptide.. Am J Physiol Endocrinol Metab.

[40] Casas S, Gomis R, Gribble FM, Altirriba J, Knuutila S, Novials A (2007). Impairment of the ubiquitin-proteasome pathway is a downstream endoplasmic reticulum stress response induced by extracellular human islet amyloid polypeptide and contributes to pancreatic beta-cell apoptosis.. Diabetes.

[41] Leighton B, Cooper GJ (1988). Pancreatic amylin and calcitonin gene-related peptide cause resistance to insulin in skeletal muscle in vitro.. Nature.

[42] Sowa R, Sanke T, Hirayama J, Tabata H, Furuta H (1990). Islet amyloid polypeptide amide causes peripheral insulin resistance in vivo in dogs.. Diabetologia.

[43] Mirzabekov TA, Lin MC, Kagan BL (1996). Pore formation by the cytotoxic islet amyloid peptide amylin.. J Biol Chem.

[44] Anguiano M, Nowak RJ, Lansbury PT (2002). Protofibrillar islet amyloid polypeptide permeabilizes synthetic vesicles by a pore-like mechanism that may be relevant to type II diabetes.. Biochemistry.

[45] Piemonti L, Calori G, Mercalli A, Lattuada G, Monti P (2003). Fasting plasma leptin, tumor necrosis factor-alpha receptor 2, and monocyte chemoattracting protein 1 concentration in a population of glucose-tolerant and glucose-intolerant women: impact on cardiovascular mortality.. Diabetes Care.

[46] Harsimran K, Singh AA, Guruvinder S, Sharda S, Vasudha S (2009). Plasma monocyte chemoattractant protein-1 as risk marker in type 2 diabetes mellitus and coronary artery disease in North Indians.. Diab Vasc Dis Res.

[47] Sell H, Dietze-Schroeder D, Kaiser U, Eckel J (2006). Monocyte chemotactic protein-1 is a potential player in the negative cross-talk between adipose tissue and skeletal muscle.. Endocrinology.

[48] Sartipy P, Loskutoff DJ (2003). Monocyte chemoattractant protein 1 in obesity and insulin resistance.. Proc Natl Acad Sci U S A.

[49] Kanda H, Tateya S, Tamori Y, Kotani K, Hiasa K (2006). MCP-1 contributes to macrophage infiltration into adipose tissue, insulin resistance, and hepatic steatosis in obesity.. J Clin Invest.

[50] Kamei N, Tobe K, Suzuki R, Ohsugi M, Watanabe T (2006). Overexpression of monocyte chemoattractant protein-1 in adipose tissues causes macrophage recruitment and insulin resistance.. J Biol Chem.

[51] Tateya S, Tamori Y, Kawaguchi T, Kanda H, Kasuga M (2010). An increase in the circulating concentration of monocyte chemoattractant protein-1 elicits systemic insulin resistance irrespective of adipose tissue inflammation in mice.. Endocrinology.

[52] Zhang N, Schröppel B, Chen D, Fu S, Hudkins KL (2003). Adenovirus transduction induces expression of multiple chemokines and chemokine receptors in murine beta cells and pancreatic islets.. Am J Transplant.

[53] Heesen M, Tanabe S, Berman MA, Yoshizawa I, Luo Y (1996). Mouse astrocytes respond to the chemokines MCP-1 and KC, but reverse transcriptase-polymerase chain reaction does not detect mRNA for the KC or new MCP-1 receptor.. J Neurosci Res.

[54] Schecter AD, Rollins BJ, Zhang YJ, Charo IF, Fallon JT (1997). Tissue factor is induced by monocyte chemoattractant protein-1 in human aortic smooth muscle and THP-1 cells.. J Biol Chem.

[55] Schecter AD, Berman AB, Yi L, Ma H, Daly CM (2004). MCP-1-dependent signaling in CCR2(-/-) aortic smooth muscle cells.. J Leukoc Biol.

[56] Westermark P, Engström U, Westermark GT, Johnson KH, Permerth J (1989). Islet amyloid polypeptide (IAPP) and pro-IAPP immunoreactivity in human islets of Langerhans.. Diabetes Res Clin Pract.

[57] Kahn SE, Andrikopoulos S, Verchere CB (1999). Islet amyloid: a long-recognized but underappreciated pathological feature of type 2 diabetes.. Diabetes.

[58] Porte D, Kahn SE (1989). Hyperproinsulinemia and amyloid in NIDDM. Clues to etiology of islet beta-cell dysfunction?. Diabetes.

[59] Clark A, Nilsson MR (2004). Islet amyloid: a complication of islet dysfunction or an aetiological factor in Type 2 diabetes?. Diabetologia.

[60] Jaikaran ET, Clark A (2001). Islet amyloid and type 2 diabetes: from molecular misfolding to islet pathophysiology.. Biochim Biophys Acta.

[61] Wang X, Xiao G, Zhang Y, Wen X, Gao X (2008). Regulation of Tcrb recombination ordering by c-Fos-dependent RAG deposition.. Nat Immunol.

[62] Marzban L, Soukhatcheva G, Verchere CB (2005). Role of carboxypeptidase E in processing of pro-islet amyloid polypeptide in {beta}-cells.. Endocrinology.

[63] Zhou C, Borillo J, Wu J, Torres L, Lou YH (2004). Ovarian expression of chemokines and their receptors.. J Reprod Immunol.

[64] Wang O, Cai K, Pang S, Wang T, Qi D (2008). Mechanisms of glucose-induced expression of pancreatic-derived factor in pancreatic beta-cells.. Endocrinology.

